# Defects in nerve conduction velocity and different muscle fibre-type specificity contribute to muscle weakness in Ts1Cje Down syndrome mouse model

**DOI:** 10.1371/journal.pone.0197711

**Published:** 2018-05-24

**Authors:** Usman Bala, Melody Pui-Yee Leong, Chai Ling Lim, Hayati Kadir Shahar, Fauziah Othman, Mei-I Lai, Zhe-Kang Law, Khairunnisa Ramli, Ohnmar Htwe, King-Hwa Ling, Pike-See Cheah

**Affiliations:** 1 Department of Human Anatomy, Faculty of Medicine and Health Sciences, Universiti Putra Malaysia, Serdang, Selangor, Malaysia; 2 Genetics and Regenerative Medicine Research Centre (GRMRC), Faculty of Medicine and Health Sciences, Universiti Putra Malaysia, Serdang, Selangor, Malaysia; 3 Department of Human Anatomy, College of Medical Sciences, Gombe State University, Gombe, Nigeria; 4 Department of Biomedical Sciences, Faculty of Medicine and Health Sciences, Universiti Putra Malaysia, Serdang, Selangor, Malaysia; 5 Department of Community Health, Faculty of Medicine and Health Sciences, Universiti Putra Malaysia, Serdang, Selangor, Malaysia; 6 Department of Pathology, Faculty of Medicine and Health Sciences, Universiti Putra Malaysia, Serdang, Selangor, Malaysia; 7 Department of Medicine, UKM Medical Centre, Jalan Yaakob Latif, Bandar Tun Razak, Cheras, Kuala Lumpur; 8 Tissue Engineering Centre, UKM Medical Centre, Jalan Yaakob Latif, Bandar Tun Razak, Cheras, Kuala Lumpur; 9 Department of Orthopaedic and Traumatology, UKM Medical Centre, Jalan Yaakob Latif, Bandar Tun Razak, Cheras, Kuala Lumpur; IGBMC/ICS, FRANCE

## Abstract

**Background:**

Down syndrome (DS) is a genetic disorder caused by presence of extra copy of human chromosome 21. It is characterised by several clinical phenotypes. Motor dysfunction due to hypotonia is commonly seen in individuals with DS and its etiology is yet unknown. Ts1Cje, which has a partial trisomy (*Mmu16)* homologous to Hsa21, is well reported to exhibit various typical neuropathological features seen in individuals with DS. This study investigated the role of skeletal muscles and peripheral nerve defects in contributing to muscle weakness in Ts1Cje mice.

**Results:**

Assessment of the motor performance showed that, the forelimb grip strength was significantly (*P*<0.0001) greater in the WT mice compared to Ts1Cje mice regardless of gender. The average survival time of the WT mice during the hanging wire test was significantly (*P*<0.0001) greater compared to the Ts1Cje mice. Also, the WT mice performed significantly (*P*<0.05) better than the Ts1Cje mice in the latency to maintain a coordinated motor movement against the rotating rod. Adult Ts1Cje mice exhibited significantly (*P*<0.001) lower nerve conduction velocity compared with their aged matched WT mice. Further analysis showed a significantly (*P*<0.001) higher population of type I fibres in WT compared to Ts1Cje mice. Also, there was significantly (*P*<0.01) higher population of COX deficient fibres in Ts1Cje mice. Expression of Myf5 was significantly (*P*<0.05) reduced in triceps of Ts1Cje mice while MyoD expression was significantly (*P*<0.05) increased in quadriceps of Ts1Cje mice.

**Conclusion:**

Ts1Cje mice exhibited weaker muscle strength. The lower population of the type I fibres and higher population of COX deficient fibres in Ts1Cje mice may contribute to the muscle weakness seen in this mouse model for DS.

## Introduction

Down syndrome (DS) is a genetic disorder due to the presence of an extra copy of the human chromosome 21 (Hsa21), a condition also known as trisomy 21 [1]. This disorder is characterised by a complex set of pathologies and several clinical phenotypes, such as intellectual disability, a characteristic set of facial features, hypotonia, cardiac defects, and anomalies associated with the immune, endocrine and digestive systems [2–4]. Individuals with DS display several forms of motor dysfunction, including gross and fine motor deficits, poor balance and motor coordination and movement variability [5,6]. In addition, these individuals have delayed motor function development, difficulties in motor planning, perceptual motor deficits and instabilities in trunk and postural control [7–9] as well as stiff joints [10]. Normal muscle strength is necessary for daily living activities and in individuals with DS, the presence of muscle weakness significantly affects their ability to perform some functional tasks [11]. Muscle weakness in individuals with DS can negatively impact on their occupational and social development [12] as well as their quality of life [13,14].

The pathological cause of muscle weakness in individuals with DS is not fully understood. Structural and functional alterations in the central nervous system (CNS) have been implicated as the main cause of muscle weakness in individuals with DS. Previous reports have suggested that there are alterations to the cerebellum, including volume reduction [15], reduction in the number of cells [16] and impaired neuronal connectivity with other structures [17]. Alterations in the cortex of individuals with DS have also been reported, including volume reduction [18] and microdysgenesia [19], decreased neuronal density, and abnormal neuronal distribution in layers II and IV [20].

Different mouse models of DS have been generated over recent years and exhibit similar phenotypes seen in individuals with DS [21]. Several motor dysfunctions, such as delayed motor skill development, poor fine motor movement and impaired motor coordination have been reported in some of these mouse models [22–24]. Hsa21 shares a conserved homology with regions of 3 mouse chromosomes, *Mmu10*, *Mmu16*, and *Mmu 17*[25]. The Ts1Cje model carries a region of *Mmu16* with approximately 85 genes homologous to those on Hsa21 [1,26]. Several studies have indicated that the Ts1Cje model exhibits some features associated with individuals with DS [26–28]. Characterisation of phenotypes in the Ts1Cje model is still underway and motor dysfunction is one feature that has not yet been fully characterised. Although motor dysfunction has been identified in other mouse models of DS, it is important to determine if the Ts1Cje mouse is also a suitable model for the study of muscle weakness. A battery of behavioural tests was first performed to determine muscle weakness in Ts1Cje mice. Subsequently, we further evaluated the nerve conduction velocity, the role of muscle fibre-types specificity and myogenic regulatory factors (MRFs) protein expression in contributing to muscle weakness in Ts1Cje mouse model of DS.

## Materials and methods

### Ethical approval, animal breeding, genotyping, and animal husbandry

This study was approved by the Institutional Animal Care and Use Committee (IACUC), Faculty of Medicine and Health Sciences (FMHS), Universiti Putra Malaysia (UPM) (Reference number: UPM/FPSK/PADS/BR-UUH/00494). Animal handling was performed in accordance with IACUC guidelines. The mice used in this study were generated by mating male Ts1Cje mice on a C57BL/6 background (obtained from Walter and Eliza Hall Institute for Medical Research, Melbourne, Australia) with wildtype (WT) C57BL/6 females. The Ts1Cje littermates were identified by tail genotyping using polymerase chain reaction (PCR) to amplify the *Grik1* gene in WT mice, while both *Grik1* and *Neo* genes were amplified in the Ts1Cje mouse as described by Sago *et al*., [26]. The disomic littermates served as the normal control (WT) for the study. Mice were kept in the mouse room facility, Genetics and Regenerative Medicine Research Centre (GRMRC), FMHS, UPM. All mice were housed under controlled temperatures with a 12:12 hour light-dark cycle. The mice were given unlimited access to standard animal feed (Altromin 1324, Lage, Germany) and clean water *ad libitum*.

### Functional assessment of motor activity

Mice at postnatal day (P)-60 to 70 consisting of both genders were subjected to a battery of behavioural studies [(n = 22 for WT (male = 7; female = 15), n = 17 for Ts1Cje (male = 10; female = 7)]. Forelimb grip strength was assessed using an automated grip meter (BIOSEB, In Vivo Research Instruments, Vitrolles, France) as described by Luca, [29]. The mice were held by the tail and gently lowered to the height, where their forelimbs were able to reach the metal mesh and were gently pulled away by their tails in the horizontal plane until their grip was broken. The maximum force generated by the mouse forelimbs was recorded (unit: Newton). The hanging wire test is another test used to evaluate the strength of the forelimb muscles. The greater the strength of the forelimb muscle, the longer the survival period (latency). The “falls and reaches method” [30] was used to evaluate the latency of a mouse to fall off a metal wire after exhaustion. Mice that exhibited an unexpected behaviour such as balancing properly on the wire and those that refused to hang by the forelimbs were excluded from the experiment. Average latency and cumulative number of falls for each mouse was computed. Motor coordination and balance was assessed as described by Deacon, [31], using a commercially available rotarod apparatus (Ugo Basile North America Inc., Collegeville, Pennsylvania, USA). The motor coordination was assessed using both fixed (4 rpm for 60 seconds) and accelerated (4–64 rpm over 120 seconds) velocities. In each case, the experiment ended when the mouse fell off the treadmill or when the total time elapsed. The trial was also halted when the mouse lost muscle coordination and clung to the rotating rod for three consecutive rotations. Each experiment was repeated three times and the average value was computed. All functional assessment tests were performed blinded with respect to the mouse genotype.

### Electrophysiological activities of the sciatic nerve

Measurement of electrophysiological activities of the sciatic nerve *in vivo* was performed as described by Schulz *et al*., [32] with minor modifications. The mice were anaesthetised with 100mg/kg body weight of ketamine and 10mg/kg of xylazine via intra-muscular injection at the forelimb [adult male mice, (P60–70), n = 14 for WT, n = 12 for Ts1Cje; ageing male mice, (P345-460), n = 6 for WT, n = 6 for Ts1Cje]. For stimulation and recording of compound muscle action potentials, a portable electromyography unit (VIASYS Healthcare, Conshohocken, Pennsylvania, USA) was used. The subdermal needle electrodes of 13 mm length (Rhythmlink International LCC, South Carolina, Columbia, USA) were placed at different positions (active electrode at the muscle belly of the gastrocnemius, reference electrode near to the tendon of the muscle with earth electrode between them). A distance of 10 mm was maintained between the active and reference electrodes. Two positions of stimulation, the distal and proximal points with a distance of 4 mm and 16 mm from the active electrode respectively were used. Sciatic nerve stimulation was performed using a constant voltage of 10 mV. The latency and the amplitude after each stimulation was computed by the VikingQuest^TM^, version 9.0 (Natus Medical Inc. Pleasanton, California, USA) and the values were recorded.

### Histochemical staining of the skeletal muscle

Skeletal muscles; quadriceps and triceps from the hindlimb and forelimb respectively were harvested from male mice at P60-70 and processed for both paraffin and frozen sections from each genotype (n = 7 per group). Paraffin-embedded sections (8 μm) of the skeletal muscles were obtained and mounted on the glass slides. The haematoxylin and eosin (H & E) (Haematoxylin (HHS16); Eosin (HT110116) Sigma- Aldrich, St. Louis, Missouri, USA) stain was used to assess the general morphology of the skeletal muscles following the manufacturer’s protocol. Frozen tissue sections (8 μm) of the skeletal muscles were obtained and mounted on superfrost slides for subsequent histochemical analysis. The ATPase (Bio-Optica, 30-30125LY, Milan, Italy) and NADH diaphorase (Bio-Optica, 30-30113LY, Milan, Italy) stains were performed according to the manufacturer’s protocol. Activity of the cytochrome *C* oxidase (COX) was assessed following the manufacturer’s protocol (Bio-Optica, 30-30115LY, Milan, Italy). Examination of the sections was performed using a bright-field light microscope (Olympus BX51, Tokyo, Japan) and images were captured through CCD digital camera attached to the microscope. Three slides were randomly selected from each sample and four images were obtained from each slide at four different quadrants for analysis of each staining. Morphological analysis of the acquired images such as cell cross sectional area (CSA) and fibre type counting were carried out using Image J software, http://rsb.info.nih.gov/ij/ (National Institutes of Health, Bethesda, Maryland, USA). In all the experiment, morphological analysis was performed single-blinded with respect to the mouse genotype.

### Western blot analysis

Skeletal muscles; quadriceps and triceps (n = 5 for Ts1Cje; n = 4 for WT), soleus and extensor digitorum longus (EDL) (n = 3 for both Ts1Cje and WT) from male adult mice (P 60–70) were harvested and homogenised in ice-cold radioimmunoprecipitation assay (RIPA) buffer (Millipore Corporation, Bedford, Massachusetts,USA) supplemented with a protease inhibitor cocktail (Calbiochem, San Diego, California, USA) and phosphatase inhibitors (Calbiochem, San Diego, California, USA). Protein extract was obtained after 30 minutes of centrifugation at 13,000 rpm, at 4°C. The protein concentration was determined by the Lowry Assay (Bio-Rad Laboratories, Munchen, Germany). About 20 μg of the total protein was denatured at 90°C for 10 minutes and subjected to 10% SDS polyacrylamide gel electrophoresis (PAGE) and then transferred onto nitrocellulose membrane. The membrane was blocked with 5% w/v BSA or non-fat dried milk in Tris- buffered saline containing 0.05% Tween-20 (TBS-T). The membrane was incubated with the following primary antibodies; MyoD (Ab16148; Abcam, Cambridge, UK; 1:1250), Myogenin (Ab124800; Abcam, Cambridge, UK; 1:400), Myf5 (sc-302; Santa Cruz Biotechnology, Inc, Santa Cruz, California, USA; 1:400) and GAPDH (Ab125247; Abcam, Cambridge, UK; 1:5000) overnight at 4°C on a shaker. The membrane was washed in TBS-T, followed by incubation in horse radish peroxidase conjugated secondary antibodies; goat anti-mouse (sc-2005; Santa Cruz Biotechnology, Inc, Santa Cruz, California, USA; 1:2500) and goat anti-rabbit (#7074; Cell Signalling Technology Inc., Beverly, Massachusetts, USA; 1:2500) following the manufacturer’s instruction for 1 hour at room temperature. Membrane was washed in TBS-T and the immunoreactivity of the proteins were detected and visualised using the chemiluminescence kit (WesternBright^TM^ Sirius^TM^, Advansta Corp, Menlo Park, California, USA). For each protein, two replicates were produced. Images were taken and pixelation analysis of the bands was performed using ImageJ (http://imagej.nih.gov/ij/) according to the standard protocol. Optical density of the bands was quantified and normalised against the loading control (housekeeping protein), glyceraldehyde 3-phosphate dehydrogenase (GAPDH) and average value from the two replicates was used for statistical analysis.

### Statistical analysis

For various behavioural and nerve conductance velocity tests, two–way analysis of variance (ANOVA) was performed to assess the effect of 2 factors (genotype and age or, genotype and gender), followed by *post hoc* Bonferroni multiple comparison test using Prism 7 (GraphPad, Software, San Diego, California, USA). A multiplicity adjusted p-value for each comparison was considered significant when <0.05. For other studies, student’s *t*-test was used to compare 2 groups/genotypes. The results were expressed as mean ± SEM and the probability values of *P* < 0.05 were considered to be statistically significant.

## Results

### Ts1Cje mice have weaker muscle strength and poor motor coordination

**Fig 1:**
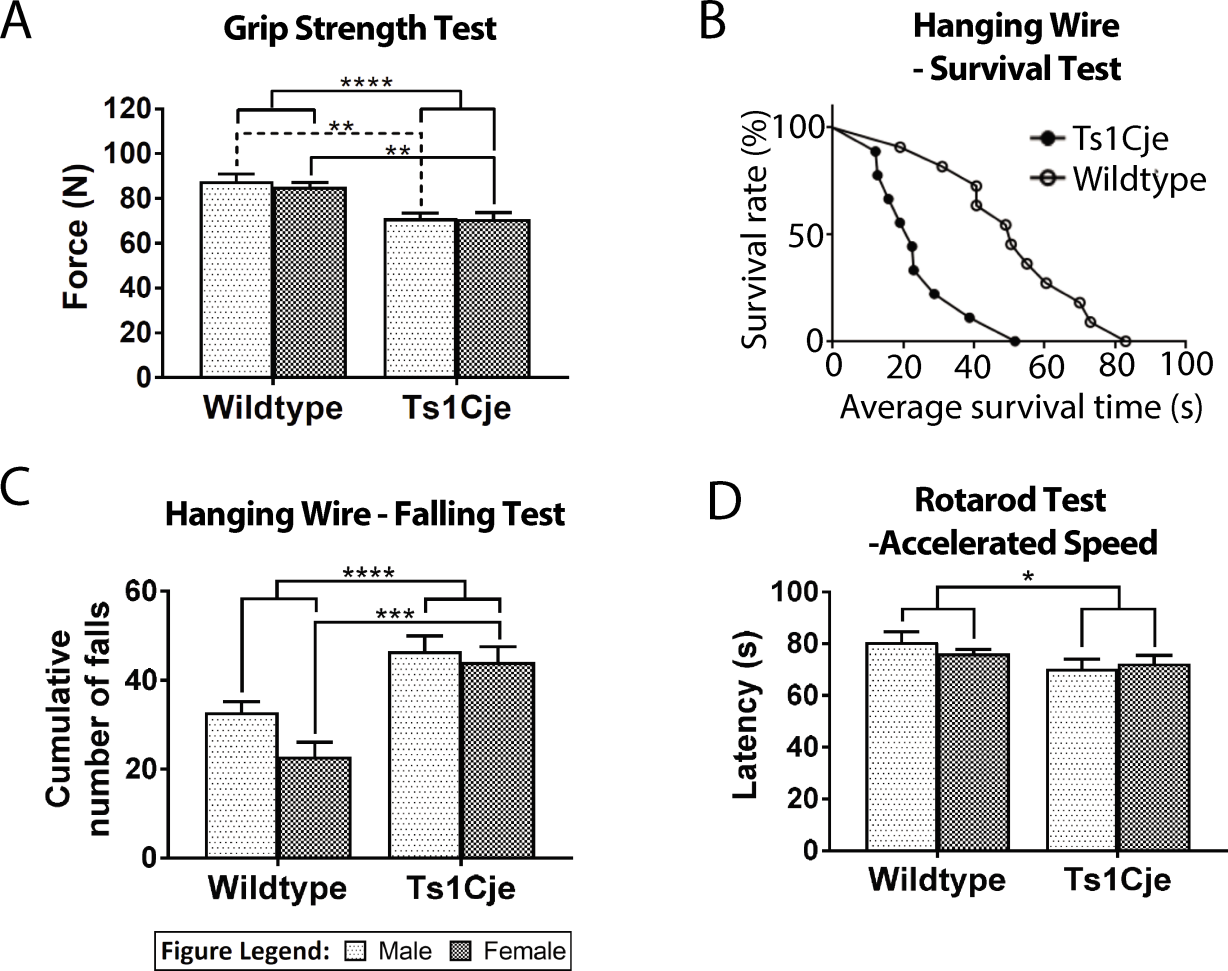
Behavioural assessment of muscle weakness in Ts1Cje mouse. Forelimb grip strength was significantly (*P*<0.0001) greater in the WT mice compared to the Ts1Cje mice for both genders (male: *P* = 0.0016; female: *P =* 0.0021) **(A).** For the hanging wire test, the survival proportion of the WT mice was significantly (*P*<0.01) greater than that of the Ts1Cje mice **(B).** Ts1Cje mice had a significantly (*P*<0.0001) greater number of falls compared to the WT mice for both genders **(C)**. At an accelerated speed of 4–64 rpm, the motor coordination of the WT mice was significantly (*P*<0.05) greater than that of the Ts1Cje group **(D).** Asterisks *, **, *** and **** denote p <0.05, 0.005, 0.0005 and 0.0001 respectively.

The grip strength meter was used to evaluate the muscle strength of the Ts1Cje mice. Forelimb grip strength was significantly greater in the WT mice (86.04±1.7N, n = 22) compared to the Ts1Cje mice (70.91±1.9N, n = 17; genotype accounts for 47.49% of total variance; F _(1,34)_ = 32.25; *P*<0.0001) (Fig 1A). Two-way ANOVA revealed no significant main effect for gender (F_(1,34)_ = 0.2715; *P* = 0.6057) for the forelimb grip strength. The grip strength of the male WT mice (85.30±3.4N, n = 7) was significantly greater than that of the male Ts1Cje mice (71.02±2.6N, n = 10; *P* = 0.0016) (Fig 1A). Similarly, female WT mice (86.39±1.9N, n = 15) had significantly stronger grip strength than the female Ts1Cje mice (70.74±2.9N, n = 7; *P* = 0.0021) (Fig 1A). For the hanging wire test, the survival proportion of the Ts1Cje mice was significantly lower compared to the WT mice (*P*<0.0001) (Fig 1B). The cumulative number of falls was significantly greater in the Ts1Cje mice (45.47±2.49, n = 17) compared to their WT littermates (26.57±2.44, n = 21; genotype accounts for 35.11% of total variance; F_(1,34)_ = 21.53, *P*<0.0001) (Fig 1C), regardless of gender. [(Male; Ts1Cje = 50.33±2.23, n = 10; WT = 24.55±4.26, n = 7; *P* = 0.0193); (Female; Ts1Cje = 43.10±3.81, n = 7; WT = 28.64±1.94, n = 14; *P* = 0.0106)]. Two-way ANOVA revealed no significant main effect for gender (F_(1,34)_ = 1.877; *P* = 0.1797) for the cumulative number of falls in hanging wire test. The relationship between the latency to fall and body weight of the mice (R = 0.72, n = 32) was not statistically significant (*P* = 0.684). The motor coordination of the mice was assessed using two-way ANOVA and there was no significant difference in the latency to fall between the WT (59.8±0.12s, n = 20) and Ts1Cje mice (58.95±0.56s, n = 17; *P* = 0.1421) at constant speed of 4 rpm. However, the WT mice (77.70±1.73s, n = 18) performed significantly greater than the Ts1Cje mice (70.54±2.39s, n = 15; genotype accounts for 15.66% of total variance; F_(1,28)_ = 5.343; *P* = 0.0284) in the latency to maintain a coordinated motor movement against the rotating rod during the accelerated speed of 4–64 rpm (Fig 1D). Further two-way ANOVA analysis revealed that, the differences in the motor coordination between the genotypes are independent of sex (F_(1,28)_ = 0.159; *P* = 0.6931) (Fig 1D).

### Male Ts1Cje mice exhibit low sciatic nerve conduction velocity

**Fig 2:**
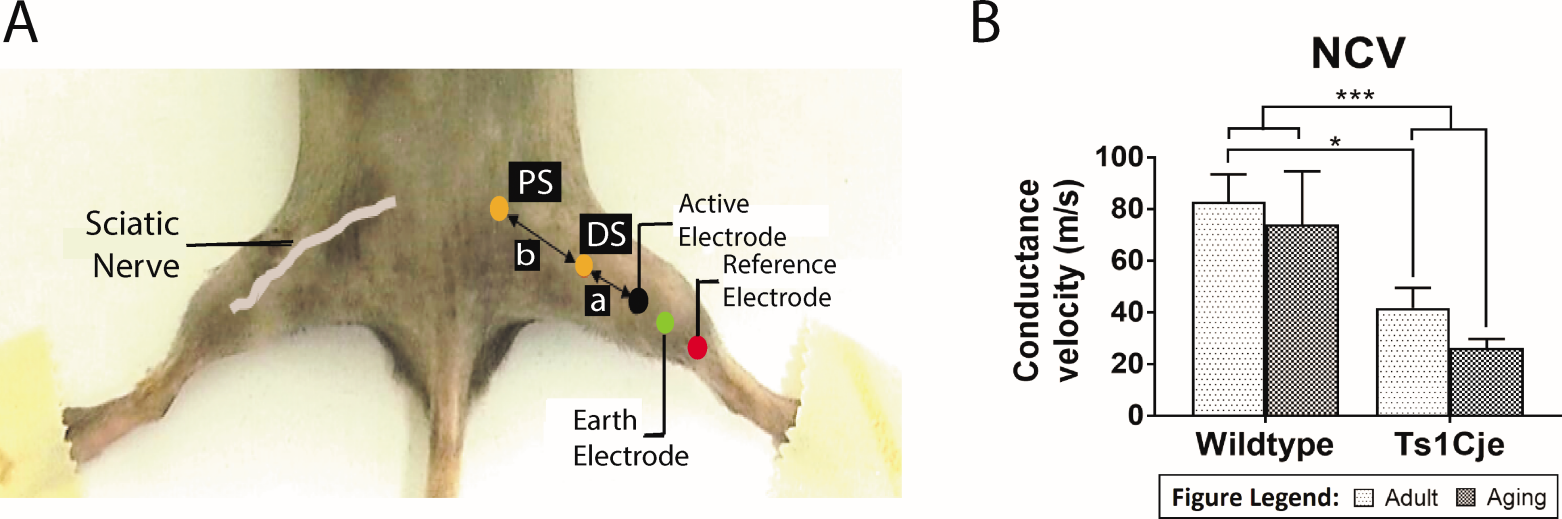
Nerve conduction velocity analysis. Measurement of electrophysiological activities of the sciatic nerve *in vivo* showing the different positions of the electrodes **(A).** The nerve conduction velocity was significantly higher in WT adult (*P* = 0.0009) but not significant in ageing WT male mice (*P* = 0.1418) as compared to the Ts1Cje male mice **(B).** Asterisks * and *** denote p <0.05, and 0.0005 respectively.

Poor motor performance of Ts1Cje led us to further investigate the health of sciatic nerve by measuring the nerve conduction velocity (NCV) as described previously (Fig 2A). The result of the *in vivo* measurement of the sciatic nerve conduction velocity in adult (but not the ageing group) group showed a significant difference between the WT and Ts1Cje male mice. In the adult mice, the NCV in the WT male mice (82.86±10.61 m/s; n = 14) was significantly higher as compared to the Ts1Cje male mice (41.76±7.76 m/s; n = 12; genotype accounts for 26.61% of total variance; F_(1,34)_ = 13.26; *P* = 0.0009) (Fig 2B). In the ageing group, post hoc Bonferroni test revealed no significant differences in the NCV between the WT (74.00±20.59 m/s; n = 6) and Ts1Cje male mice (26.19±3.55 m/s; n = 6; *P* = 0.1418) (Fig 2B**)**. We further assessed whether aging has contributed to the low conduction velocity. Two-way ANOVA analysis revealed no significant main effect on NCV due to age between male adult and aging groups (F_(1,34)_ = 1.001; *P* = 0.3241).

### Male Ts1Cje mice have lower population of type I muscle fibres and higher proportion of COX-negative fibres

**Fig 3:**
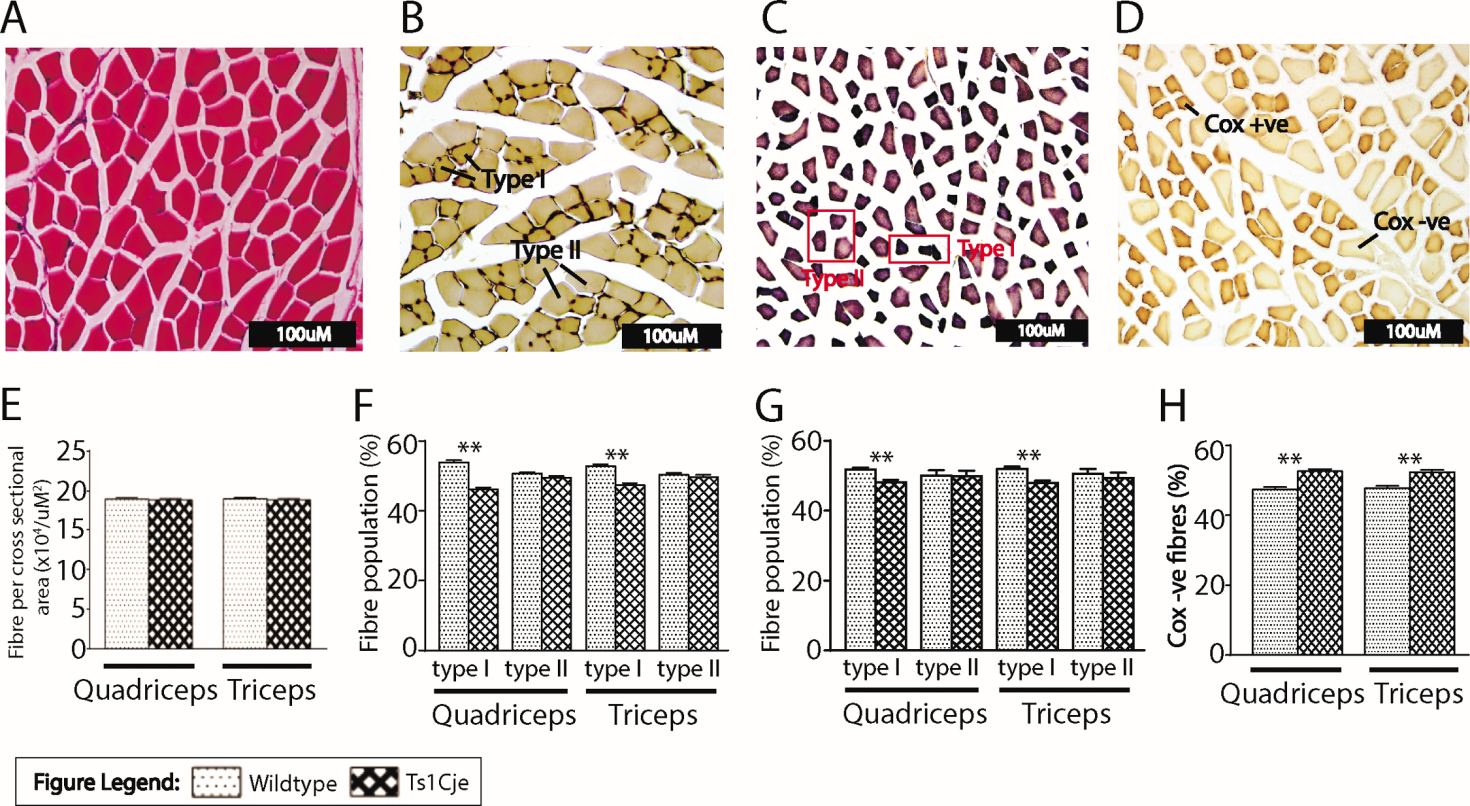
**Histomorphological assessment of the skeletal muscles in Ts1Cje male mice using haematoxylin & eosin stain (A)** ATPase stain **(B)** NADH diaphorase **(C)** and Cytochrome *c* oxidase (D). Morphometric analysis of the H & E sections **(A)** showed no significant difference in the cross sectional area of the fibres in both quadriceps (*P* = 0.695) and triceps (*P* = 0.676) of Ts1Cje as compared with the WT male mice **(E).** Analysis of the ATPase **(B)** and NADH diaphorase **(C)** stained sections indicated that the population of type I fibres was significantly (*P*<0.001) higher in the quadriceps and triceps of WT male mice than that of Ts1Cje male mice both in ATPase **(F)** and NADH diaphorase **(G).** The percentage population of COX-negative fibres **(D)** in Ts1Cje male mice was significantly higher (*P*<0.01) in both in quadriceps and triceps as compared with the WT male mice **(H).**

Cross sections of the skeletal muscles stained with haematoxylin and eosin showed no morphological differences between the Ts1Cje and WT male mice (Fig 3A). Similarly, no significant differences were observed in the cross sectional area (CSA) of the fibres both in quadriceps (*P* = 0.695) and triceps (*P* = 0.676) between the Ts1Cje and WT male mice (Fig 3E).

Muscle fibres are generally classified into type I and type II based on their reactivity with the myofibrillar enzymes (ATPase and NADH diaphorase). Comparing to type II fibres, the type I fibres stained more deeply in blue and beige with ATPase and NADH diaphorase, respectively (Fig 3B and 3C). Analysis of the ATPase sections showed that, the percentage population of type I fibres was significantly higher in the quadriceps (WT = 51.40 ± 0.586 (53.9%); Ts1Cje = 43.97 ± 0.616 (46.1%); n = 250; *t* = 1.802; *P*<0.001) and triceps (WT = 50.08 ± 0.591 (52.7%); Ts1Cje = 44.87 ± 0.668 (47.3%); n = 248; *t* = 1.040; *P*<0.001) (Fig 3F) of WT male mice as compared with that of Ts1Cje male mice. Fibre type analysis from the stained sections of NADH diaphorase (Fig 3C) also revealed a significantly higher population of type I fibres in quadriceps (WT = 42.30 ± 0.627 (51.8%); Ts1Cje = 39.46 ± 0.652 (48.2%); n = 242; *t* = 0.144; *P*<0.01) and triceps (WT = 43.16 ± 0.651 (52.0%); Ts1Cje = 39.92 ± 0.602 (48.0%); n = 249; *t* = 0.809; *P*<0.01) (Fig 3G) of WT male mice to that of Ts1Cje male mice. However, both histochemical stains showed that the population of type II fibres of quadriceps (*P* = 0.3) and triceps (*P* = 0.3) of Ts1Cje male mice was comparable to WT littermates (Fig 3F and 3G).

Mitochondrial function was further assessed using cytochrome *C* oxidase (COX) stain (Fig 3D). Muscle fibres that react positively with complex IV stained deep beige or brown, thus indicating the present of normal mitochondrial activity in the fibres. In contrast, fibres that showed negative activity to the complex IV stained white or pale beige signifying a deficiency of complex IV of the electron transport system in the fibres [33] (Fig 3D). The result from the COX stain revealed a significantly higher population of the COX-negative fibres in the quadriceps (Ts1Cje = 51.92 ± 0.639 (52.6%); WT = 46.78 ± 0.676 (47.4%); n = 152; *t* = 4.451; *P*<0.01) and triceps (Ts1Cje = 50.48 ± 0.651 (52.3%); WT = 45.97 ± 0.735 (47.7%); n = 126; *t* = 4.595; *P*<0.01) of Ts1Cje male mice as compared with the WT (Fig 3H).

### MRFs protein expression in skeletal muscle

**Fig 4:**
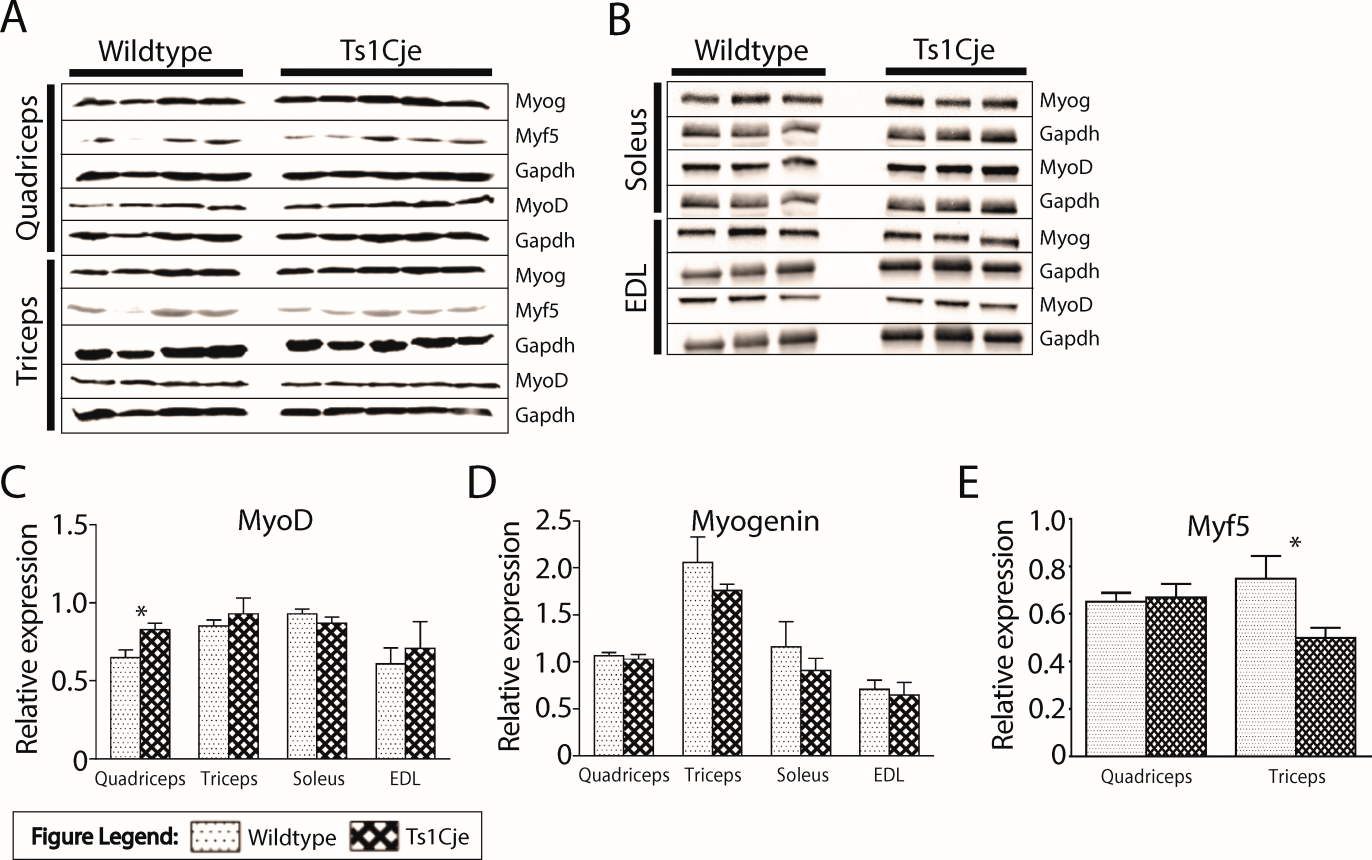
**The protein expression of myogenic regulatory factors (MRFs) markers in quadriceps and triceps (A) Soleus and EDL (B).** MyoD expression was found to be significantly (*P*<0.05) upregulated in Ts1Cje quadriceps **(C).** There was a reduction trend of the expression levels of myogenin in the muscles screened in Ts1Cje male mice but it was statistically insignificant **(D).** Myf5 was significantly (*P*<0.05) downregulated in the Ts1Cje triceps **(E).**

The myogenic regulatory factors (MRFs) are involved in muscle formation and differentiation of the myoblasts to myocytes. The development is regulated and controlled by myogenin, Myf5 and MyoD [34]. Structural and functional activities of the skeletal muscle may be explained by the efficiency of myogenesis process. Moreover, the amount of these proteins in the skeletal muscle may also determine the degree of post-natal myogenesis. We further assessed whether there is altered expression of these proteins in the Ts1Cje skeletal muscle (Fig 4A and 4B). MyoD was not significantly different in the skeletal muscles of WT and Ts1Cje male mice except for its significant upregulation in Ts1Cje quadriceps [quadriceps: WT = 0.65 ± 0.05, Ts1Cje = 0.83 ± 0.04, *P* = 0.026; triceps: WT = 0.85± 0.04, Ts1Cje = 0.93 ± 0.10, *P* = 0.493; soleus: WT = 0.93 ± 0.03, Ts1Cje = 0.87 ± 0.038, *P* = 0.3365; EDL: WT = 0.61 ± 0.10, Ts1Cje = 0.71 ± 0.17, *P* = 0.6712] (Fig 4C). Myogenin expression was not significantly different in all the skeletal muscles for both genotypes [quadriceps: WT = 1.07 ± 0.03, Ts1Cje = 1.03 ± 0.05, *P* = 0.4820; triceps: WT = 2.06 ± 0.27, Ts1Cje = 1.76 ± 0.07, *P* = 0.273; soleus: WT = 1.16 ± 0.27, Ts1Cje = 0.91 ± 0.13, *P* = 0.4567; EDL: WT = 0.71± 0.10, Ts1Cje = 0.65 ± 0.13, *P* = 0.6978] (Fig 4D). Similarly, Myf5 expression was not significantly different except for significant downregulation in Ts1Cje triceps (*P*<0.05) [quadriceps: WT = 0.65 ± 0.04, Ts1Cje = 0.67 ± 0.06, *P* = 0.805; triceps: WT = 0.75 ± 0.10, Ts1Cje = 0.50 ± 0.04, *P*< 0.05] (Fig 4E).

## Discussion

Hypotonia is one of the features seen in individuals with DS and yet there is no clear understanding of what causes muscle weakness. Many studies implicate that CNS contributes to muscle weakness in individuals with DS. Several neuroanatomical defects affecting the CNS and neuromuscular junction have been reported in individuals with DS [35,36], and in *Drosophila* homologs of Hsa21 [37]. This study aimed to further understand the cause(s) of the muscle weakness, by investigating the nerve conduction velocity and skeletal muscles of the Ts1Cje mouse model for DS.

Behavioural assessments from the present study have supported the suitability of the Ts1Cje mice in the muscle weakness related study, as this mouse model for DS recorded a significantly lower muscle strength compared with the aged matched WT mice. Similarly, due to their weaker muscle strength, the Ts1Cje mice could not stay in the hanging position for long and they fell down more frequently compared to the WT mice, resulting in higher cumulative number of falls which was found to be significant compared to the WT mice. Results from this study are in line with other studies on Ts65Dn and TgDyrk1A transgenic mice [24,38].

Ts1Cje mice also exhibit impairment in balance and motor coordination, but less severe as compared with other mouse models for DS. Our data shows that the Ts1Cje mice acquired the skill to remain on the stationary rod after a few trial sessions and they performed at the rates nearly similar to the WT mice at a constant speed of 4 rpm. On the other hand, the Ts65Dn mice showed delay in acquiring the skill to stay on the stationary rod and their ability to remain on the rod differs significantly from their control littermates [24]. In the present study, the Ts1Cje mice maintained an intact motor coordination until 30 rpm but the Ts65Dn showed significant deficits in motor coordination at a lower speed of 15 rpm [24]. Also, the Tc1 mouse model displays a different degree of impairment across all tasks, including the static and accelerating rotarod tests. In spite of extensive training sessions across several days, the Tc1 model did not show any sign of improvement, as they could not maintain intact motor coordination even at 4 rpm [22].

In the present study, the poor performance of Ts1Cje in the rotarod is further supported by the substantial decrease in NCV of Ts1Cje sciatic nerve. Reduced NCV can arise from thinner myeline sheath as well as axonal dysfunction [39]. It is also important to take into consideration that other factors may also influence the NCV such as length of internodal Schwann cells and the growth of Schwann cells [40,41]. These cellular mechanisms warrant further investigation in the model of DS.

MRFs play an important role in the process of myogenesis and any alteration in their expression will affect a particular stage or the entire process of myogenesis, leading to defective muscle formation. With limited studies on expression of the MRFs in mouse models for DS and in individuals with DS, their roles warrants further investigation. Alteration in fascicular architecture, organisation and histochemical characteristics of the skeletal muscles may contribute to muscle weakness. The skeletal muscles of the Ts1Cje male mice showed a normal muscle morphology as compared to the WT male mice. No significant fibre morphology difference was observed between the two genotypes. However, the population of type I fibres in both quadriceps and triceps was significantly lower in Ts1Cje male mice as compared with WT male mice and this has been consistent in both ATPase and NADH analyses. Type I muscle fibres have higher resistance to fatigue than type II fibres and this may possibly explain the reason for the poor performance of Ts1Cje mice during the hanging wire test and rotarod test. The Ts1Cje mice could not endure the hanging position and fell down within few seconds of their hanging. Similarly, these mice could not sustain the treadmill rotation and they lost motor coordination much earlier as compared to the WT. On the other hand, the ability of the WT mice to stay longer in the hanging position and perform excellently during the rotarod test may indicate the higher degree of endurance in these mice and it may be associated with higher population of the type I fibres as observed in quadriceps and triceps. Previous study in Ts65Dn mice revealed no significant differences in the population of fibre types between the two genotypes in soleus using immunofluorescence studies [42].

The population of the COX deficient fibres was significantly higher in Ts1Cje male mice as compared with the WT male mice and the result of this study further supported the general understanding of the mitochondrial defects in individuals with DS [43–46]. A defective mitochondrial function in the quadriceps of individuals with DS was found *in vivo* [46] and the expression of COX2 protein was also found to be significantly lower in the soleus of Ts65Dn mice [42]. There is an association between the number of the mitochondrial deficient fibres and the severity of the disease, as the higher the number of the affected fibres the greater the severity of the disease [47]. The Ts1Cje male mice has higher number of COX negative fibres and further analysis showed that, the differences in the proportion of these fibres between the two groups did not reach threshold level and therefore this condition (mitochondria dysfunction) in Ts1Cje mice may be classified as mild type [48]. In addition, the lower population of the mitochondrial deficient fibres in the Ts1Cje male mice may result in low ATP generation and may be associated with increased intracellular levels of reactive oxygen species [49] leading to oxidative stress and hus may warrant further investigation.

## Conclusion

Ts1Cje mouse model for DS have weaker muscle strength, poorer balance and impaired motor coordination compared with their control littermates and hence this model is suitable to be used for DS research related to muscle weakness. The lower motor conduction velocity of the peripheral nerve has impacted the motor performance of Ts1Cje. The expression of MRFs did not provide a conclusive evidence on their role in causing muscle weakness in Ts1Cje male mice. However, Ts1Cje male mice have significantly lower population of type 1 muscle fibres and higher number of mitochondrial deficient fibres and this may explain their poor motor performance.
